# Ionic Liquid Crystals as Chromogenic Materials

**DOI:** 10.3390/ma17184563

**Published:** 2024-09-17

**Authors:** Andreia F. M. Santos, João L. Figueirinhas, Madalena Dionísio, Maria H. Godinho, Luis C. Branco

**Affiliations:** 1 LAQV-REQUIMTE, Department of Chemistry, NOVA School of Science and Technology, NOVA University of Lisbon, 2829-516 Caparica, Portugal; afm.santos@fct.unl.pt (A.F.M.S.); madalena.dionisio@fct.unl.pt (M.D.); 2 CeFEMA and Department of Physics, Instituto Superior Técnico, University of Lisbon, 1049-001 Lisbon, Portugal; joao.figueirinhas@tecnico.ulisboa.pt; 3 i3N/CENIMAT, Department of Materials Science, NOVA School of Science and Technology, NOVA University of Lisbon, 2829-516 Caparica, Portugal; mhg@fct.unl.pt

**Keywords:** ionic liquid crystals, chromogenic materials, photochromism, electrochromism, thermochromism

## Abstract

Ionic liquid crystals (ILCs), a class of soft matter materials whose properties can be tuned by the wise pairing of the cation and anion, have recently emerged as promising candidates for different applications, combining the characteristics of ionic liquids and liquid crystals. Among those potential uses, this review aims to cover chromogenic ILCs. In this context, examples of photo-, electro- and thermochromism based on ILCs are provided. Furthermore, thermotropic and lyotropic ionic liquid crystals are also summarised, including the most common chemical and phase structures, as well as the advantages of confining these materials. This manuscript also comprises the following main experimental techniques used to characterise ILCs: Differential Scanning Calorimetry (DSC), Polarised Optical Microscopy (POM) and X-Ray Powder Diffraction (XRD). Chromogenic ILCs can be interesting smart materials for energy and health purposes.

## 1. Introduction

The search for greener technologies to be implemented in cities and communities is one of the Sustainable Development Goals projected in Agenda 2030 by the United Nations. In this context, smart materials, and their stimuli-responsive ability to environmental variations [1], have been explored over the years, driven by the interest in developing novel ergonomic molecules with optimised performance compared to traditional systems [2,3]. They also provide paths to design sustainable technologies, contributing positively to the global environment. In fact, several industrial applications have demonstrated the advantages of using these materials, particularly in construction, soft robotic mechanisms, drug delivery and bioremediation, among other fields. Moreover, piezoelectric ceramics, electroactive polymers, shape-memory alloys, magneto- and electrorheological fluids, as well as chromogenic materials, were reported as exhibiting sensing functions [1,4,5,6]. The latter are capable of changing their colouration upon one or more external stimuli [7]. For example, photo-, electro-, thermo-, piezo- and halochromic molecules show colour modifications induced by varying the light, electrical potential, temperature, pressure or pH, respectively. In parallel, the limited availability of fossil energy resources, and the consequent pollution associated with this stream, has caused an intensive demand for renewable clean sources, leading to novel energy storage and conversion devices [8,9].

**Figure 1:**
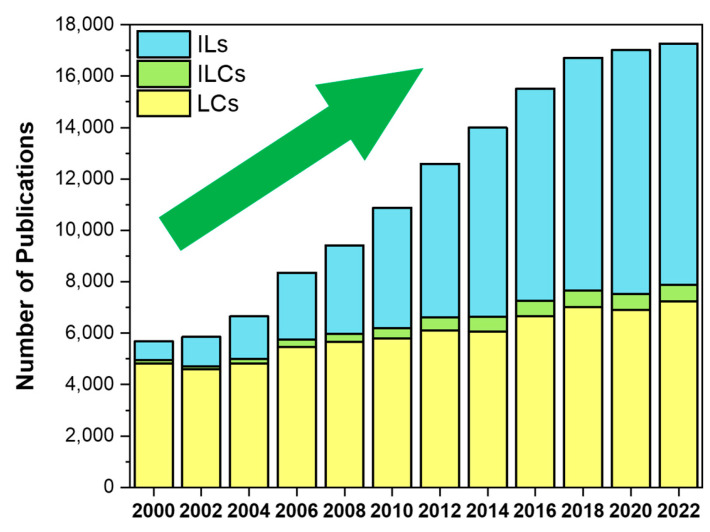
Number of papers published per year about ionic liquids, liquid crystals and ionic liquid crystals, revealing the growing interest in the third topic driven by the increasing attention on ionic liquids. Data from Web of Science™, using “ionic liquids”, “liquid crystals” and “ionic liquid crystals” as query keywords.

Task-specific ionic liquids (ILs) emerged as promising candidates for several applications, including smart materials [10,11,12] and electronics [13,14,15], mainly due to the possibility of predicting their final characteristics by the unique combination of cations and anions. Additionally, the search for novel technologies with improved performance has recognised that liquid crystals (LCs) can enhance the materials’ efficiency [16]. These soft materials have also proven their relevance for different purposes: from soaps [17] and cosmetics [18,19] to biomimetic materials [20] and energy applications [21,22,23]. Furthermore, the combined features of ionic liquids with liquid crystals originate ionic liquid crystals (ILCs), a class of materials of growing interest capable of being ruled by the molecular design to modulate their properties. In fact, although ILs and LCs have been intensively studied in the last decades, few papers have been published on the merging of these two topics, as illustrated in Figure 1.

Thus, this review is motivated by the recent attention to this class of advanced materials, providing insights on ionic liquids and liquid crystals along with a guide to basic characterisation techniques. Moreover, the confinement of mesomorphic materials into nanosized structures is also covered, as well as chromogenic ionic liquid crystals, particularly the photo-, electro- and thermochromic ones, which have been poorly explored in the literature. Future perspectives about this subject are also included. For an overview of the structures prone to be mesogenic, the papers published by K. Binnemans [24], H. Ohno [25], S. Laschat [26] and their respective co-workers are worth noting. The state-of-the-art general functional LCs [27] and ILCs [28] has also been recently addressed.

## 2. Liquid Crystals

Isotropic liquids and crystalline solids are the two most studied phases of condensed matter, yet are differing in their order: in crystals, the molecules obey a three-dimensional ordered arrangement, while, in liquids, this is not observed. Usually, a crystal exhibits positional and orientational order, as well as minimal mobility, constraining the molecules to occupy specific sites in a matrix and leading them to align their molecular axes to a specific direction. Contrarily, in a liquid state, the molecules diffuse randomly with no positional and orientational order [29,30,31]. However, some materials have more than one single-phase transition, not only within crystalline phases, as in the case of polymorphic materials [32], but between solid to isotropic liquid, involving mesophases.

In 1888, the first liquid crystal phase was reported by Friedrich Reinitzer [33], an Austrian botanist and chemist at the German University in Prague, Czechoslovakia. He observed that when he melted a cholesterol-like substance (cholesteryl benzoate), it first became a cloudy liquid that cleared up when the temperature increased. Upon cooling, the phenomenon appeared to be reversible, as the liquid turned blue before the final crystallisation. To solve this enigma, Reinitzer contacted Otto Lehmann, a German physicist and crystallographer who observed optical anisotropy on the translucid liquid phase of Reinitzer’s cholesterol esters. He attributed this behaviour to the existence of elongated molecules aligned, proposing the designation of “fluid crystals” and “liquid crystals” [34]. Nonetheless, only in the second decade of the 20th century, liquid crystals were referred as a new state of matter, intermediate or mesomorphic, between solid crystals and ordinary liquids [35].

**Figure 2:**
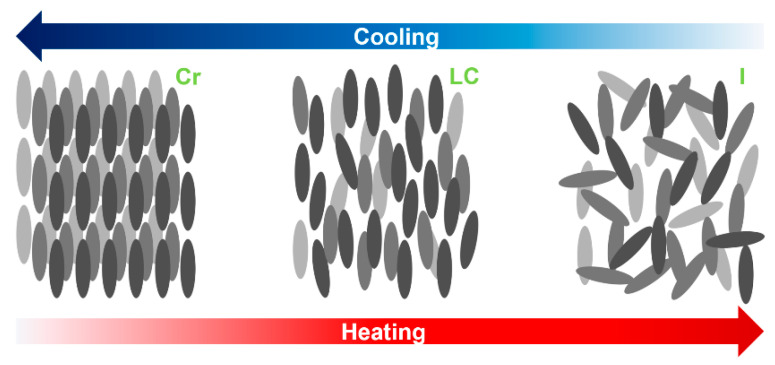
Phase transformations of calamitic molecules and their respective arrangement in terms of long-range order for crystal (Cr), liquid crystal (LC) and isotropic (I) phases.

In fact, liquid crystals (LCs) are anisotropic compounds, exhibiting characteristics of both crystalline solids and isotropic liquids [29,36,37,38,39]. Figure 2 schematises the phase transformations and the respective molecular arrangement in terms of long-range order. As mentioned before, these fluids exhibit long-range orientational order, but no positional order, along with birefringence, fluidity and the ability to self-assemble, as well as other specific properties that highlight the relevance of these materials in modern science and industry [18,27,40,41,42]. The generally cloudy appearance addressed to this state of matter is responsible for light scattering, which is associated with the formation of domains. Moreover, the birefringence shown by LCs translates into the light propagation in the anisotropic material that experiences two indices of refraction, as in crystals. Hence, the observation of a birefringent material between crossed polarisers reveals colourful patterns and diverse textures.

**Figure 3:**
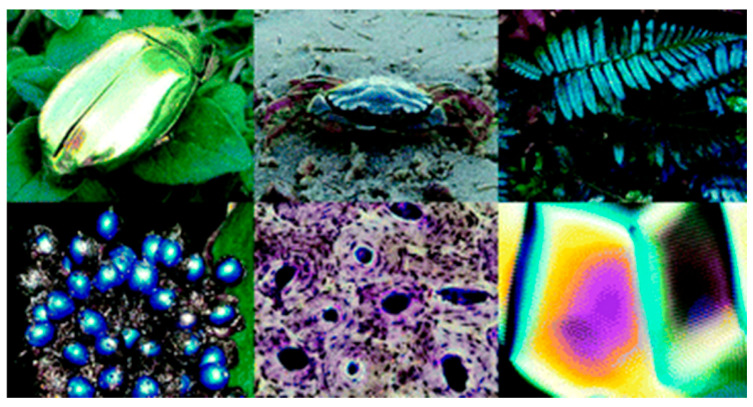
Examples of liquid crystals in living matter. Image retrieved from [43].

Liquid crystals are ubiquitous in nature, particularly in plants and animals (Figure 3) [43]. It is curious to mention that DNA (deoxyribonucleic acid), which encodes the genetic information of most living matter, possesses liquid crystalline organisations with different structures [44,45], supporting also the self-assembling of chromatin (complex of DNA and proteins found in eukaryotic cells) [43,46]. Additionally, several studies focused on the LC phases of virus suspensions revealed distinct mesophases associated with the single-stranded DNA helicoidally wrapped by proteins [47,48,49,50,51].

Cellulose and chitin are the two most abundant biopolymers, having, each one, a wide range of applications [52,53,54]. Whereas the former polysaccharide is mainly produced by plants and found in trees, fruits and leaves, but specially in wood, the latter can be extracted from crustaceans, being the main constituent of the arthropods’ exoskeleton. They both display liquid crystalline behaviour. In fact, this peculiar feature is responsible for the camouflage strategy of some beetles, known as iridescent beetles [55]. Structural iridescence, addressed to the cellulose nanocrystals, is also present in leaves and fruits to either turn the plants more attractive to pollinators or to protect them from herbivores [56,57]. Silk is another example of living matter where it is possible to detect mesomor-phism, specifically in solution or in the fibroins present in the early duct portion of the major silk-producing gland in *Nephila clavipes* spider and *Bombyx mori* silkworm [58,59]. Furthermore, the LC nature of collagen was demonstrated in vitro (solution and films) [60], as well as in vivo (bones, tendons, cornea and fish armour) [61].

However, different synthetic structures have been developed. Three classic examples of these compounds are 5CB (4-cyano-4′-pentylbiphenyl), PAA (*p*-azoxyanisole) and E7, which is a mixture of different components [62,63], including 5CB. Indeed, the vast popularity of 5CB is due to the fact that it was the first molecule synthesised for display devices near room temperature [64]. PAA also played a role in the development of such devices, having a LC range from 118 °C to 135 °C [65].

## 3. Classification of Liquid Crystals

**Figure 4:**
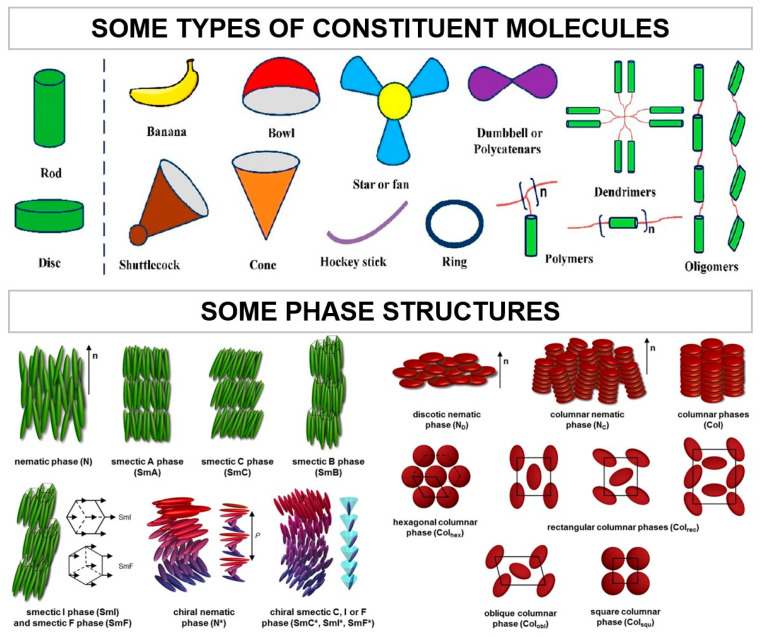
Classification of liquid crystals according to their type of constituent molecules and phase structure. Images adapted from [24,73].

Liquid crystals can be classified in terms of their thermodynamic genesis, type of constituent molecules and phase structure. Examples of the latter two are displayed in Figure 4.

Thermodynamic-wise, mesophases emerging upon temperature or concentration/solvent variations are designated as thermotropic or lyotropic liquid crystals, respectively. In this context, thermotropic liquid crystalline phases, which are normally associated to first order transitions, occur in a certain temperature range, by a set of thermal processes, from crystal to liquid crystal and isotropic liquid, where pressure and concentration are constant [29]. Thus, thermotropic liquid crystals can present multiple mesophases, being, in general, reversible. If a certain transition is irreversible and only arises upon heating or cooling, it is called monotropic [66,67].

On the other hand, lyotropic mesophases appear in solution when mesogenic units are dissolved in a suitable solvent, meaning that the liquid crystallinity of a certain material and its stability are controlled by the concentration of the solution at pressure and temperature constants [68,69,70]. Generally, lyotropic mesophases are formed by amphiphilic molecules, i.e., molecules containing a hydrophilic polar head, that can interact with water through hydrogen bonding, and a hydrophobic non-polar tail, which is repelled by water [37,68,71]. These molecules, when contacting with a solvent, for instance, water, tend to arrange themselves, exposing one part to the environment. In this case, the increasing concentration leads to the formation of micelles, where the hydrophobic tails assemble together, facing the hydrophilic heads to the water. For lower concentrations, the amphiphilic molecules are distributed randomly in the solvent. It is worthwhile noting that some materials are able to form both thermotropic and lyotropic mesophases, being designated as amphotropic [72].

In general, thermotropic mesophases are relevant in electro-optic devices, including so-called liquid crystal displays (LCDs) and temperature and pressure sensors, while lyotropic systems have proven to be of great interest biologically and play an important role in living systems [71].

Regarding the type of constituent molecules, mesophases are promoted by molecules with anisotropic shape, either elongated, disk-like or banana-shaped, originating calamitic, discotic or banana-shaped/bento-core liquid crystals, respectively [29,74,75]. These molecules consist of a central rigid core (normally aromatic) and a flexible tail (often aliphatic groups), which can form structures with low molecular weight or high, as elastomers and polymers [38].

As previously referred, different molecular structures, associated with the chemical properties and molecular geometry of LCs, lead to distinct liquid crystalline phases, ranging from nematic (N) to smectic (S), columnar (Col) and cubic (Cub) phases [76]:

- **Nematic:**

The word “nematic” comes from the Greek word for thread and is addressed to the type of defects that are commonly observed in these phases [29]. Moreover, the molecular alignment in a one-dimensional structure, in which one or two molecular axes are oriented parallelly to another, pointing in the same direction and not layered, results in molecules that are free to rotate or slide past one another. Indeed, this mesophase is the one presenting the highest fluidity and lowest viscosity, similar to those of isotropic liquids. Nematic phases can emerge in both calamitic and discotic molecules. Furthermore, when a certain mesogenic molecule contains at least one chiral carbon, a peculiar nematic phase arises, namely the cholesteric (N*) phase, whose name derives from the fact that this phase was first observed on cholesterol derivatives [77]. N* mesophases possess a very specific phase structure, resembling a helix, as the molecules are orientated in an helicoidal manner around a perpendicular axis (optical axis). Contrarily to nematic phases, whose higher mobility translates into a less cloudy liquid with lower viscosity, the cholesteric ones tend to be opaquer.

- **Smectic:**

**Figure 5:**
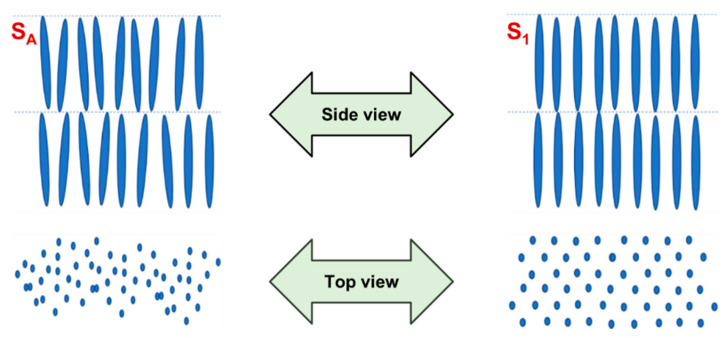
Side and top view illustrating how the molecules are organised in a disordered smectic A (S_A_) phase and an ordered smectic (S_1_) phase.

Similar to the nematic phases, the origin of the name smectic is related to a Greek word that translates to soap-like. In fact, Friedel [78] did not recognise the existence of more than one smectic phase, but he noticed that it had a soap-like appearance. These mesophases present a layered structure with well-defined interlayer spacings, as well as a translational order in the direction perpendicular to the layers, being commonly designated as lamellar phases. The fact that the interactions between layers are weak when compared to the lateral forces between the molecules allows layers to slide over one another relatively easily, promoting an increased viscosity of S phases relative to the nematic ones [79]. Moreover, smectic liquid crystals are more ordered than the nematics, in which their lamellar molecular organisation can have several degrees of translational and non-orientational orders, being responsible for the formation of different mesophases, such as the S_A_, S_B_, S_C_, S_F_ and S_G_ phases [28]. The referred smectic phases differ from each other in layer formation and in the existing order inside the layers. In particular, S_A_ and S_C_ phases are known for displaying disordered smectic phases, meaning that there is no regular arrangement of the molecular centres. Additionally, the molecules of the S_A_ phases are perpendicular to the plane of the layers and have no positional order within the layers, while the molecules of the S_C_ phases are tilted inside the layers, exhibiting a non-zero angle alignment with the normal. This angle is temperature dependent and decreases to zero upon heating, originating a S_A_ phase if the liquid crystal exhibit both phases. On the other hand, other smectic phases exhibit ordered layers, where, in general, the molecules are placed in an organised way, maintaining a structure of the molecular centres typically arranged hexagonally. Figure 5 schematises the difference between the S_A_ and a smectic phase with ordered layers.

- **Columnar:**

As the name suggests, in this mesophase, the molecules are organised in a columnar structure, giving origin to two-dimensional lattices with different arrangements depending on the columns’ distribution, promoting hexagonal, tetragonal, rectangular, oblique or square phases.

- **Cubic:**

One of the less common mesophases to appear, cubic phases exhibit structures with micellar lattice units or complex interwoven networks.

For a thermotropic calamitic system having multiple mesophases, upon heating, the degree of order decreases with the transformation between phases. In this context, firstly, positional order is partially maintained in the smectic C and A phases. Then, the positional order is lost within the entrance into the nematic phase that only exhibits orientational order. Finally, when all the liquid crystalline order disappears, the isotropic phase is reached [77,80].

## 4. Ionic Liquid Crystals

- **Ionic liquids:**

In 1914, P. Walden reported the first ionic liquid successfully synthesised: ethylam-monium nitrate [81]. He observed that this unusual combination of an organic cation and inorganic anion, creating an asymmetric salt, had a melting point of ~12 °C, being substantially lower than the traditional inorganic salts, whose melting is much higher (>500 °C) [82]. The explanation to the depletion of the melting point is associated to the size and non-symmetry of the salt’s components, leading to an unstable crystalline network with an inferior lattice energy and, thus, easier to melt [83]. Conventionally, it was established that ionic liquids (ILs) are organic salts with a low melting point, normally below 100 °C. Some are even liquid at room temperature, being denominated RTILs (room temperature ionic liquids). Since ionic liquids are salts, the wise pairing of their cations and anions allows to modulate the final properties of the compound [82,84,85,86]. This fine tuning, extremely relevant in several fields, is addressed by the huge diversity of ions that can be incorporated in ionic liquids.

In the last decade of the 20th century, ILs have gained popularity in a wide range of applications and the studies involving this class of materials have increased exponentially since then. For this reason, in 2007, R. D. Rogers et al. [87] divided ionic liquids into three different generations. The first generation is associated to the origin of ionic liquids as organic solvents, taking advantage of their tuneable physical properties and being relevant in fields that require the design of solvents [88,89,90,91,92]. Furthermore, the second generation, in which the unique physicochemical characteristics of the cation and anion pair enable the architecture of new functional tailor-made materials that retain the core desired features of an IL, has opened doors to a wide range of applications, aiming to enhance the performance of the already commercialised technologies [93,94,95,96,97]. Finally, the most recent class of ionic liquids, the third generation, combines the aforementioned physical and chemical properties with biological functions to improve drug formulations in the pharmaceutical industry [87,98,99,100]. In fact, ionic liquids have proven their efficiency in this field, particularly in what concerns the suppression of polymorphism, one of the major drawbacks of the pharmaceutical industry [101,102,103].

- **Ionic liquid crystals:**

**Figure 6:**
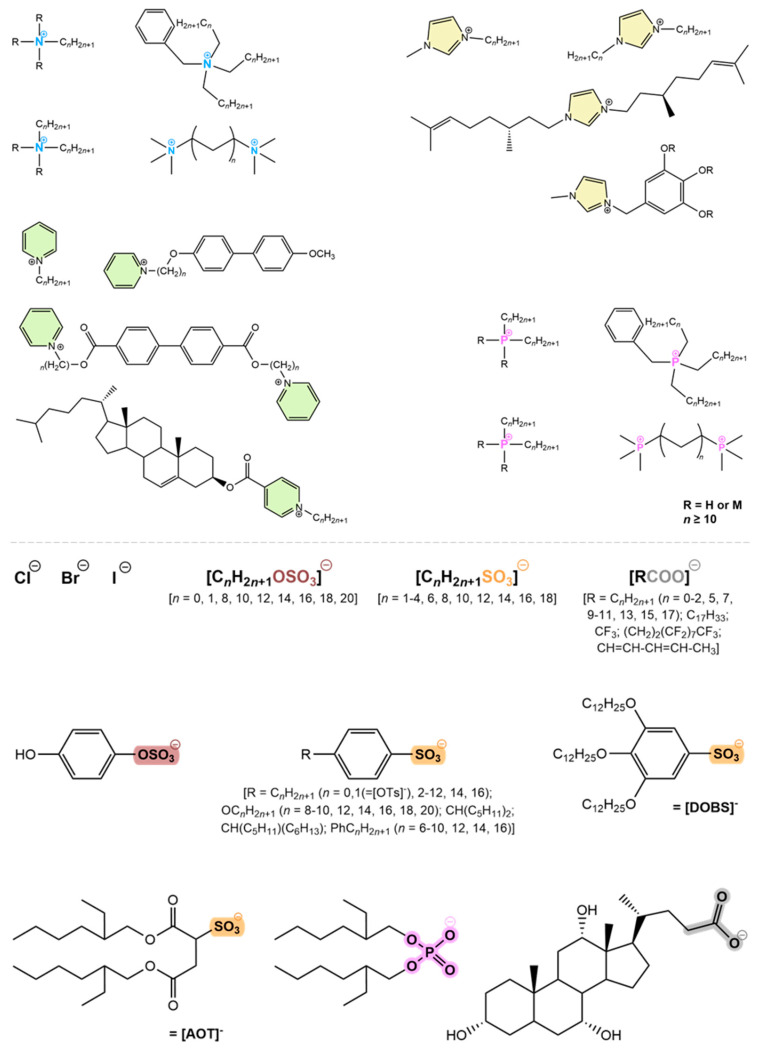
Main families of cations and anions responsible for originating low molecular mass ionic liquid crystals. Cations: ammonium (blue), imidazolium (yellow), pyridinium (green) and phosphonium (pink). Anions: halides (Cl^−^, Br^−^, I^−^), sulphates (brown), sulfonates (orange), phosphates (pink) and carboxylates (grey).

Within the framework of the second generation, several task-specific ionic liquids were developed [104]. The combination of the synergetic properties of an ionic liquid and a liquid crystal, i.e., when a certain material has liquid crystalline properties and is simultaneously ionic, originated a new class of advanced materials known as ionic liquid crystals (ILCs) [26,28,105,106]. Although the first ionic liquid crystals were described in 1938 [105], the exploration of these materials has increased during the last two decades driven by the interest in ionic liquids. Most reported ILCs are based on an aromatic ring coupled to a long alkyl chain, resulting in rod-like structures that promote lamellar phases. On the contrary, discotic shapes, which are less common in ionic liquids with liquid crystalline features, tend to give rise to columnar phases. In order to design novel ionic liquid crystals, it is important to select the appropriate alkyl chain length. Smaller sizes hinder the formation of liquid crystal phases, leading to crystalline materials, while longer chains promote increased mobility, potentially disrupting the long-distance orientational order, a critical feature to take into account when molecular self-assembly is envisaged. Thus, sizes between C_6_ and C_18_ are suitable for forming liquid crystals [106,107,108,109]. In fact, K. Binnemans and co-workers [24] provided a comprehensive overview of the mesogenic structures associated with thermomesomorphism (Figure 6). These materials have potential applications in sustainable energy storage and conversion devices [110,111,112,113] due to their ability to order and self-assemble into diverse arrangements that facilitate multidimensional ion conductive pathways [9,114], high cohesion energy to the electrode surface [115], multiple types of cation–anion interactions (electrostatic, Van der Waals interactions, conventional and non-conventional hydrogen bonds) [116,117,118], as well as high electrochemical stability and enlarged voltage window [119,120].

- **Thermotropic and lyotropic ionic liquid crystals:**

As for liquid crystals, ionic liquid crystals can be thermotropic, lyotropic or, ultimately, amphotropic. Comparing the number of studies involving the first two topics, while thermotropic ILCs are well documented in the literature, exhibiting mainly lamellar phases, lyotropic systems are less addressed. Despite the lower number of publications, multiple phase structures were reported for lyotropic ionic liquid crystals. For instance, a hexagonal phase was observed for the aqueous solutions based on the methylimidazolium surfactants [C_10_MiM][NO_3_] [121], [C_14_MiM][Br] [122] and [C_16_MiM][Acr] [123], in which the latter also displays cubic phases [123]. In contrast, metal alkanoates dissolved in water exhibit smectic mesophases [124,125,126]. These phases were also identified for protic pyridiniums [127] and for anionic surfactant carboxylates [128] in the presence of various solvents. Notably, for choline laurate ([Ch][Lau]), in dimethyl sulfoxide (DMSO), a transition from hexagonal to a lamellar phase was detected upon the addition of α-tocopherol [128]. Furthermore, ternary phase diagrams obtained from mixtures of a non-ionic surfactant in water with either [C_4_MiM][BF_4_] or [C_4_MiM][PF_6_] revealed different mesophases, namely lamellar, hexagonal and cubic [129].

- **Confinement of ionic liquid crystals:**

The search for ways to optimise the materials’ performance pointed out confinement as a strategy to either tune the physical state of the guest, stabilising the material in a phase different from the one exhibited by the bulk, or even to imprint a new behaviour. Particularly, when the confining matrix is porous with a pore size of below the critical size for crystal growth, crystallisation of the molten guest can be avoided, becoming a supercooled liquid, which vitrifies upon further cooling [130]. Furthermore, if the pore dimensions interfere with the length scale for collective motion, an acceleration of the dynamics of the pore core population may occur compared to the bulk, while the surface-anchored population undergoes a slowing down of its mobility due to interactions with the host matrix [131,132]. Therefore, the behaviour upon confinement is the interplay between finite size and surface effects.

If a liquid-like phase, such as a mesophase, is stabilised inside a porous matrix, it is possible to obtain a solid-like material with potential to be used in a wide range of applications, including photonic devices [133,134,135] and electronics. In this context, depending on the alignment and order adopted by the guest within the confining matrix [136], mostly induced by the interaction with the pore wall [137], different outcomes can be found. For ionic liquid crystals, confinement into nanosized structures allows the modification of their phase behaviour [138,139,140,141], as well as the widening/enhancement of their optical and electric properties relative to bulk [140,142], characteristics also common to other mesomorphic materials [137,143,144,145].

## 5. Characterisation of Ionic Liquid Crystals

Typically, there are three main techniques involved in the study of liquid crystals and, therefore, ionic liquid crystals: (i) Differential Scanning Calorimetry (DSC) to map the eventual liquid crystalline temperature range, (ii) Polarised Optical Microscopy (POM) to confirm the presence of birefringence and fluidity of the mesophase and (iii) X-Ray Powder Diffraction (XRD) to determine the phase structure.

- **Differential Scanning Calorimetry:**

DSC is a thermodynamic technique widely used to monitor the phase transformations of a material through variations in the heat flow/heat capacity against an empty pan. It requires a low amount of sample, in the order of 4 mg. Thermal events, such as the release and absorption of energy or changes in the heat capacity of substances, can be studied accurately and quickly by DSC.

These calorimetric measurements are carried out in function of temperature and time [146], allowing to heat and cool a certain material at a preset rate to provide accurate information about its physical and energetic properties [147] and to classify it as crystalline, amorphous, liquid, polymorphic, among others. For crystalline materials, during heating, the three-dimensional network is broken down due to the energy that the system receives, which leads to less ordered structures, engraved on the thermograms as endothermic peaks [148,149]. The transition from a crystal to an isotropic liquid is commonly known as melting and the area under the melting peak (T_m_) represents the total amount of heat absorbed upon this first order transition [147], i.e., the latent heat, or the enthalpy, of the conversion between states [149]. The estimation of such physical quantity allows to calculate the crystallisation degree through the ratio of its value by the melting enthalpy of the full crystalline structure. Similar to melting, crystallisation is also a first order transition with latent heat associated. However, in this phenomenon, a kinetic component is addressed to the nucleation and growing of crystals [150]. Contrarily to melting, during crystallisation, the material’s order degree increases, releasing thermal energy, while converting to a lower energy state [151]. This generates an exothermic peak, whose maximum corresponds to the crystallisation temperature (T_c_), with the associated enthalpy that, once again, can be estimated by the area under the peak [147]. On the other hand, the glass transition is absent of latent heat, being characterised by a small change in the heat capacity [148], which translates into a discontinuity in the heat flow over a range of temperatures [152]. The step from the baseline, where it is possible to extract the temperatures of the onset (T_g_onset_), midpoint (T_g_midpoint_) and endset (T_g_endset_) of the glass transition, is dependent on the rate at which the sample is heated or cooled [152,153].

**Figure 7:**
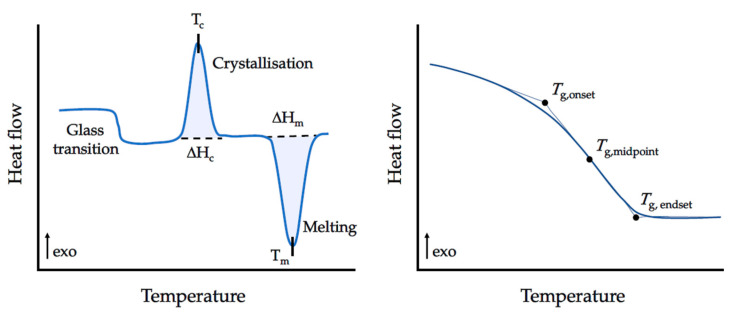
A schematic DSC scan, showing the main transitions that can be detected by this calorimetric technique. Images adapted from [153].

Hence, the main transformations are registered as exothermic (crystallisation), endothermic (melting) or by a discontinuity of the heat capacity curve (glass transition) [148,152]. Figure 7 includes an example of a thermogram, comprising these transformations and a zoom of the T_g_ with the different temperature locations highlighted.

Considering that thermotropic mesophases can emerge on heating, on cooling or on both runs, in general, transitions associated with this state appear as exothermic or endothermic peaks. Normally, thermograms of mesomorphic materials present multiple peaks related to the different transformations. The enthalpy associated to these thermal events allows for the correlation of phase transitions with their degree of ordering, leading to preliminary conclusions about their structural organisation. For instance, a small enthalpy involved in the conversion from LC to isotropic liquid suggests a disordered mesophase.

- **Polarised Optical Microscopy:**

The use of optical microscopy to visualise living matter dates back about four centuries, when A. Van Leeuwenhoek perfected the equipment and popularised it for the observation of bacteria, red blood cells and sperm [154]. Nowadays, it is still a very useful technique to observe different types of samples. Particularly, the use of polarised light has increased in the last few decades, providing comprehensive insights into different specimens in a broad range of scientific fields (e.g., mineralogy, biology, medicine, polymer chemistry and liquid crystals) [155]. For crystalline materials, POM provides information related to their local anisotropy, which is a consequence of molecular order, and optical properties, as refraction (birefringence) and absorption (dichroism) [156].

As mentioned, a polarising microscope is designed to visualise samples at the microscale mainly due to their optically anisotropic character. In order to fulfil this task, the microscope must be equipped with a polariser and an analyser, where the first component is placed before the specimen to polarise the light that illuminates the observation field, and the second one is located between the objective rear aperture and the eyepieces or camera port [156]. These two polarisers can be crossed in relation to each other, being designated as crossed polarisers. Moreover, the observation of samples can be performed between cross-polars in transmission or reflection modes.

**Figure 8:**
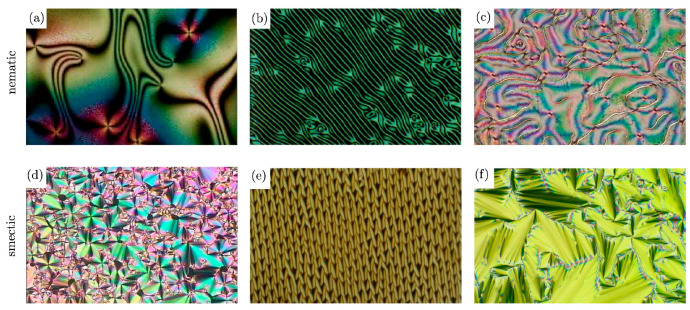
Typical textures of nematic and smectic mesophases: (**a**–**c**) Schlieren textures with singularity points and disclination lines, (**d**–**f**) textures with focal conics. Image adapted from [39].

The identification of liquid crystalline phases by POM involves their magnified view between two glass slides. For thermotropic mesophases, the sample is inserted in a temperature-controlled stage, whereas lyotropic phases can be observed at room temperature. The mesophase can be detected through the presence of fluidity and the analysis of the textures. If the polarisers are crossed at 90° and no sample is between, a black field is observed. For isotropic liquids, the polarised light remains unaffected by the sample and no light passes through the analyser, leading also to a black field. Nonetheless, for anisotropic specimens, the light is not extinguished and a birefringent texture can emerge [38]. This texture, derived from the defects that exist either as localised points and minor misorientations in the structure or as extensive structural discontinuities [157], can provide relevant insights on the mesophase structure [158]. Usually, nematic liquid crystals present a Schlieren thread-like texture, while smectic phases exhibit focal conics, as depicted in Figure 8.

Therefore, it is possible to determine the phase structure by POM observations. However, for the cases where the texture detected is not common, the rule of phases [159], also known as the rule of miscibility, can allow the type of mesophase to be extrapolated through the construction of a complete phase diagram between the unknown and the reference compound. According to this rule, if two liquid crystals were placed in contact with each other and if they are miscible in all proportions, it is possible to conclude that both exhibit the same LC phase. On the contrary, if they are not miscible in all proportions, nothing can be concluded.

- **X-Ray Powder Diffraction:**

In order to further assess the physical state of the material, XRD analysis is useful to determine the long-range structural organisation, providing precise information about structures, phases and crystal orientations [160]. Crystals are three-dimensional arrays of atoms, whose molecules possess fixed positions that are repeated in space by three non-coplanar vectors. Since x-rays are electromagnetic radiation of small wavelengths with dimensions similar to the binding distances, crystals act as a diffraction graft for the incident beam [161].

**Figure 9:**
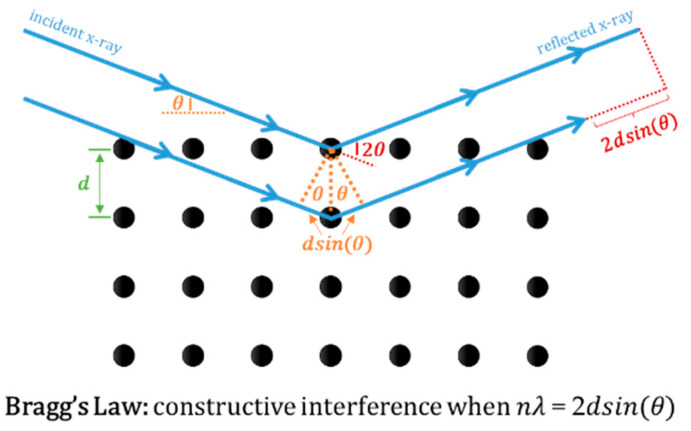
Diagram of Bragg’s Law.

Diffractometers are based on three constituents: a cathode x-ray tube, a sample holder and a detector for the diffracted rays. The first is responsible for the generation of x-rays through heating a filament that produces electrons to bombard the sample. Before reaching the material, the x-rays are filtered by foils or crystal monochromators to produce monochromatic radiation and collimated to concentrate [160]. When x-rays interact with the sample and are diffracted from the crystalline lattice, if the pathlength is equivalent to an integer multiple of the radiation wavelength, constructive interference is formed and scattering peaks are observed, corresponding to the specific incidence angles that are equal to the scattering ones. This is obeyed by Bragg’s Law (*nλ* = 2*dsin*(*θ*), Figure 9), which relates the wavelength of electromagnetic radiation (λ) to the diffraction angle (*θ*) and the interplanar lattice spacing (*d*) in a crystalline sample [162]. Moreover, when the sample rotates inside of the diffractometer, the reflected beam is at angle *θ*, while the detector is positioned at 2*θ* to collect the diffracted rays. Then, these rays are transformed into diffraction peaks and converted further into *d*-spacings to identify the substance structure by comparing these spacings with standard reference patterns [160]. Usually, data are acquired at 2*θ* from 5° to 70° [160], being divided in small (2*θ* < 5°, Small-Angle X-Ray Scattering, SAXS) and large (2*θ* > 5°, Wide-Angle X-Ray Scattering, WAXS) angles [163].

From Bragg’s Law, and as the wavelength of electromagnetic radiation is constant during the XRD experiment, it is inferred that lower *θ* values correspond to larger lattice spacing, being the reason why interlamellar distances in LC emerge at lower 2*θ* values relative to crystals. In fact, this technique is particularly relevant for the characterisation of liquid crystalline materials [164], as it allows the determination of the mesophase structure. For disordered smectic phases, S_A_ and S_C_, only one intense sharp peak appears at small angles, associated with long-range orientational order, while ordered lamellar phases also exhibit lower intensity peaks at large angles. Another parameter to be considered is the layer spacing, which for the regular S_A_ phase is identical to the full molecular length [71]. Additionally, crystallite size can also be estimated from the XRD assays, through Scherrer’s Law $\left(D=\frac{K\lambda }{\beta cos(\theta )}\right)$ [165]. This equation correlates the Scherrer constant (K) with the wavelength of the applied x-ray beam (λ) and the full width at half maximum of the peak (FWHM, β) for a specific diffraction angle (*θ*). Even though K is directly associated with the particle shape, ranging from 0.62 to 2.08, the most commonly used value is 0.9 [166].

It is worthwhile to refer that another type of disordered phases can be found in ILCs, namely plastic crystalline phases [167]. Plastic crystals, contrary to the perfect crystalline order of an ideal crystal, possess some degree of internal disorder [168,169], giving rise to the so-called rotator phases between the fully crystalline phase and the molten liquid [170]. This plastic crystal behaviour, exhibited by a diversity of materials, has also been reported for ionic crystals [171,172,173,174] and ionic liquids [170], hypothesis also raised for the ionic liquid crystal [C_16_-2-Pic][Br] [141,175], since many of their constituent moieties are long alkyl chains, which are able to retain rotational disorder in the crystalline phase [176,177,178,179]. Moreover, the local internal disorder in plastic crystals is reflected in the x-ray data by simpler diffractograms when compared to those of the associated fully crystalline phase, with a predominance of Bragg peaks at low scattering angles due to the loss of local order induced by the rotational degrees of freedom [173].

## 6. Applications of Ionic Liquid Crystals as Chromogenic Materials

**Figure 10:**
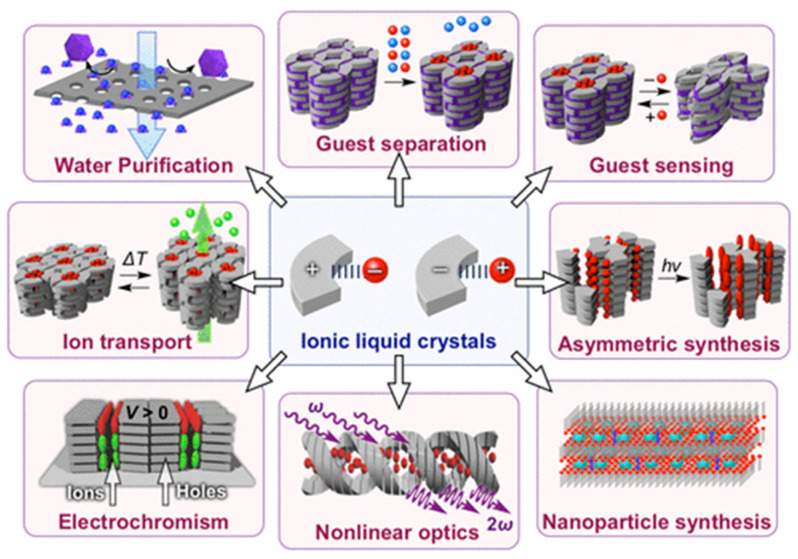
Examples of applications involving ionic liquid crystals. Image retrieved from [28].

In the last few decades, several ionic liquid crystals have been developed as promising functional soft materials, benefiting from the synthesis design, as their properties can be tuned by the wise pairing of the cation and anion. In fact, ILCs have paved the way in different areas of materials science, particularly in gas adsorption [180], extraction [128], water purification [181], lubricants [182], solar cells [183], electrolytes [110], electromechanical actuators [184], among others [18,28,111]. Figure 10 summarises some of these applications.

**Figure 11:**
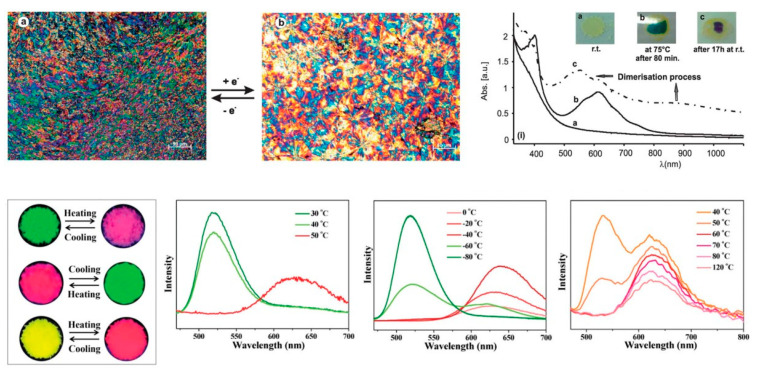
Electro- and thermochromism found in ILCs for [(C_1_)_2_BPyr][DOBS]_2_ (**top**) and [C*_n_*VIM]_m_[MnCl_x_Br_y_] (**bottom**), respectively. Images adapted from [186,192].

Regarding materials that exhibit sensing functions, the chromogenic ones are capable of changing their colouration upon one or more external stimuli. For example, photo-, electro-, thermo-, piezo- and halochromic molecules show colour modification induced by varying the light, electrical potential, temperature, pressure or pH, respectively. However, most of the papers published about chromogenic ionic liquid crystals have been focused on electro- and thermochromism. Table 1 comprises some structures already known and their respective mesophase and colour change, while Figure 11 depicts two examples of the referred phenomena.

**Table 1:** Some photo-, electro- and thermochromic ionic liquid crystals and their respective colour change.

	Compound	Liquid Crystalline Phase	Colour Change	Reference
**Photochromism**	[(C_12_ImC_1_)_2_Azo][Br]_2_	Smectic A phase	*n.d. ^a^*	[185]
	[(C_14_ImC_1_)_2_Azo][Br]_2_	Smectic A phase	*n.d. ^a^*	[185]
	[(C_10_ImC_2_O)C_1_Azo][Br]	Smectic A phase	*n.d. ^a^*	[185]
	[(C_12_ImC_2_O)C_1_Azo][Br]	Smectic A phase	*n.d. ^a^*	[185]
	[(C_16_ImC_6_O)C_1_Azo][Br]	Smectic A phase	*n.d. ^a^*	[185]
**Electrochromism**	[(C_1_)_2_BPyr][DOBS]_2_	Hexagonal columnar phase	Yellow → Blue	[186]
	[(C_8_Ph)_2_BPyr][NTf_2_]_2_	Smectic A phase	Colourless → Green	[187]
	[(C_10_Ph)_2_BPyr][NTf_2_]_2_	Smectic A phase	Colourless → Green	[187]
	[(C_14_Ph)_2_BPyr][NTf_2_]_2_	Smectic A phase	Colourless → Green	[187]
	[(C_14_)(C_2_CF_3_)BPyr][NTf_2_]_2_	Smectic X phase *^b^*	Colourless → Violet	[188]
**Thermochromism**	[C_10_-4-(SC_12_-ODZ)-Pyr][Br]	Smectic A phase	Yellow → Red	[189]
	[C_12_-4-(SC_12_-ODZ)-Pyr][Br]	Smectic A phase	Yellow → Red	[189,190]
	[C_14_-4-(SC_12_-ODZ)-Pyr][Br]	Smectic A phase	Yellow → Red	[189]
	[C_16_-4-(SC_12_-ODZ)-Pyr][Br]	Smectic A phase	Yellow → Red	[189]
	[C_1_-3-(C_7_F_15_-ODZ)-Pyr][I]	Smectic A phase	Yellow → Red	[191]
	[C_4_VIM]_m_[MnCl_x_Br_y_]	Hexagonal columnar phase	Green → Red *^d^*	[192]
	[C_8_VIM]_m_[MnCl_x_Br_y_]	*n.d. ^c^*	Red → Green *^d^*	[192]
	[C_12_VIM]_m_[MnCl_x_Br_y_]	Smectic A phase	Yellow → Red *^d^*	[192]
	[(C_1_)_2_BPyr][DOBS]_2_	Hexagonal columnar phase	Yellow → Blue	[186]

The following list is organised as: **attributed name** = *published name* = IUPAC name. **[(C*_n_*ImC_1_)_2_Azo][Br]_2_** = *1d-e* = 4,4′-bis(N-alkylimidazole-methyl)azobenzene dibromide (alkyl: *n* = 12 (*n*-dodecyl) or 14 (*n*-tetradecyl)); **[(C*_n_*ImC_2_O)C_1_Azo][Br]** = *2b-c* = 1-alkyl-10-(2-(4-((4-methylphenyl)diazenyl)phenoxy)ethyl)-imidazolium bromide (alkyl: *n* = 10 (*n*-decyl) or 12 (*n*-dodecyl)); **[(C_16_ImC_6_O)C_1_Azo][Br]** = *3e* = 1-hexadecyl-12-(2-(4-((4-methylphenyl)diazenyl)phenoxy)hexyl)- imidazolium bromide; **[(C_1_)_2_BPyr][DOBS]_2_** = *MV^2+^(DOBS)_2_* = 1,1′-dimethyl(4-pyridin-4-ylpyridinium) di-[3,4,5-tris(dodecyloxy)benzenesulfonate]; **[(C_n_Ph)_2_BPyr][NTf_2_]_2_** = *n-NTf_2_* = 1,1′-bis(4-alkyl-phenyl)-[4,4′-bipyridine]-1,1′-diium di-[bis(trifluoromethylsulfonyl)amine] (alkyl: *n* = 8 (*n*-octyl), 10 (*n*-decyl) or 14 (*n*-tetradecyl)); **[(C_14_)(C_2_CF_3_)BPyr][NTf_2_]_2_** = *1.2bp14* = 1-(3,3,3-trifluoropropyl)-1′-tetradecyl-[4,4′-bipyridine]-1,1′-diium di-[bis(trifluoromethylsulfonyl)amine]; **[C_n_-4-(SC_12_-ODZ)-Pyr][Br]** = *IIa-d* = 1-alkyl-4-[5-dodecylsulfanyl-(1,3,4-oxadiazol-2-yl)]pyridinium bromide (alkyl: *n* = 10 (*n*-decyl), 12 (*n*-dodecyl) 14 (*n*-tetradecyl); 16 (*n*-hexadecyl); **[C_1_-3-(C_7_F_15_-ODZ)-Pyr][I]** = *5a* = 3-[5-perfluoroheptyl-(1,2,4-oxadiazolyl)]-1-methylpyridinium iodide; **[C_n_VIM]_m_[MnCl_x_Br_y_]** = *[C_n_VIM]Mn_1, 2 or 3_* = 1-alkyl-3-vinylimidazolium manganese complex (alkyl: *n* = 4 (*n*-butyl), 8 (*n*-octyl) or 12 (*n*-dodecyl); anion: x = 2, m = y = 1 or m = x = y = 2). *^a^* No specific colour modification was mentioned *^b^* Smectic X phase is an unidentified smectic phase. *^c^* The structure of the mesophase was not determined. *^d^* The colours are also fluorescent.

- **Photochromism:**

Photochromism in liquid crystals has been widely explored in the literature [193,194], as these materials are known for having light-driven responses. However, only few examples cover ILCs. In this context, organic salts based on azobenzene derivatives were reported as photochromic ionic liquid crystals, being synthesised in the *trans* conformation [185]. Upon UV light irradiation, all materials undergo a *trans–cis* photoisomerisation process that reverses with visible light. It should be noted that the high temperature at which the liquid crystalline phase occurs (>100 °C) impaired the study of the photoisomerisation and the observation of colour change in this state [185]. Furthermore, X. Chen et al. [195] described a luminescent lyotropic liquid crystal with UV-induced photochromism, whose matrix is based on an amphiphile ionic liquid crystal (1-dodecyl-3-methylimidazolium bromide, [C_12_MiM][Br]) and a protic ionic liquid (ethylammonium nitrate) doped with an europium complex (Eu(DBM)_3_BQ) containing two ligands (dibenzoylmethane and biquinoline). This allowed for the preparation of a multicoloured lyotropic LC with improved photophysical properties to design novel photoluminescent materials, shifting from red (complex) or green (biquinoline) to different colourations.

- **Electrochromism:**

Electroactive species can exhibit simultaneously different properties, among them electrical conductivity, electron and charge transfer abilities, as well as electrochromism. In that sense, one building block commonly used to incorporate novel ionic functional materials is the cation 4,4′-bipyridinium, also designated as viologen. Several studies have been published addressing this structure [196,197,198,199,200,201]. S. Asaftei and his team [186] prepared three different ionic liquid crystals based on viologen units and realised that applying electrical potential impacted, apart from the material’s oxidation state, the molecular arrangement in the structure of the mesophase. Moreover, only [(C_1_)_2_BPyr][DOBS]_2_ was capable of varying its colouration, being, as will be addressed later, a multi-stimuli responsive material with electrochromic and thermochromic responses. On the other hand, selecting bistriflimide as anion of a certain viologen core allowed for the visualisation of a peculiar colour change: from colourless to green [187]. These organic salts, although having a cation with different chain sizes, exhibited a strong electrochromic behaviour, shifting, reversibly, in a fraction of a second in the mesophase [187]. Indeed, the performance enhancement of such optical devices, either in terms of stability, durability or switching rate [202,203], is one of the major advantages of using a liquid crystal in their preparation, as already observed for other non-ionic materials [204,205]. Furthermore, the group of G. Saielli [188] explored different non-symmetrically substituted polyfluorinated bipyridinium and bent-symmetrically substituted dialkyl-oxadiazolylbipyridinium bistriflimide salts, allowing the synthesis of polymesomorphic compounds, even though the former family was the one displaying electrochromism.

In another approach, the electrochromism of a luminescent liquid crystalline dye derived from cholesterol was explored in neat conditions, dispersed into a room temperature ionic liquid ([C_2_MiM][NTf_2_]) and into a gel ([C_2_MiM][NTf_2_] mixed with polymethylmethacrylate), switching, in all cases, from orange to red [206]. The advantage of the latter strategy in comparison to the other two is that the gel composite also provides a mechanical stimuli response [206], opening doors to a wide range of new applications.

- **Thermochromism:**

Thermotropic mesophases coupled with thermochromism have been described for several ionic liquid crystals. Since the early 2000s, structures derived from oxadiazolylpyridinium cations were stated as possible structures exhibiting thermochromic changes between yellow to red [189,190]. In the first study [189], varying the size of the alkyl chain, from C_10_ to C_16_, did not impact the structure of the mesophase nor the arise of the red colouration, which was found to be reversible on cooling. However, the anion plays an essential role, as it can either endorse or surpass the appearance of a new colour. In fact, among the four different anions, only the cation conjugated with a halide, more specifically bromide, bent the ideal conditions in terms of conjugation distances in the conformation of the main chain, which is a critical point in the assumed charge transfer thermochromic mechanism [190]. Furthermore, F. Lo Celso et al. [191] studied the mesomorphic behaviour of several highly fluorinated 1,2,4-oxadiazolylpyridinium salts and observed that, once again, the incorporation of a halide promoted thermochromic properties. In this context, all fluorinated pyridinium iodides exhibited a reversible colour change, although only [C_1_-3-(C_7_F_15_-ODZ)-Pyr][I] was classified as ILCs. Interestingly, the emergence of the bilayer smectic phase occurs at similar temperatures as the change to red colouration.

On the other hand, combining metals with halides in one moiety can open doors to a wide range of colours by small modifications at molecular level. In 2021, a manuscript [192] reporting the development of multi-stimuli responsive ionic liquid crystals correlated the impact of having cations with different alkyl chain lengths coupled with a metallic complex, [MnCl_x_Br_y_]^m−^, to the colour spectrum displayed by the materials. While [C_4_VIM]_m_[MnCl_x_Br_y_] and [C_12_VIM]_m_[MnCl_x_Br_y_] changed, respectively, from green to red and yellow to red upon heating at 50 °C, the material with intermediate length shifted from red to green on quench cooling under a liquid nitrogen environment. Moreover, this thermochromic mechanism, ascribed to the structural transition from the tetrahedral complex [MnCl_2_Br_2_]^2−^ to the octahedral species [MnCl_2_Br]^−^, is reversible and associated to the rise of the mesophase.

Finally, as previously referred, thermochromism was also found in an ionic liquid crystal containing methyl viologen [186]. This bipyridinium derivative has demonstrated its potential as a chromogen, being, normally, associated with electrochromism [196,197,198,199,200,201]. Nonetheless, heating up to the onset of the liquid crystalline phase promoted the appearance of the same colouration observed by the authors in the electrochromic studies [186].

## 7. Conclusions and Future Perspectives

This review delves into the development, characterisation and application of chromogenic ionic liquid crystals (ILCs). The suitable combination of the liquid crystals (LCs) and ionic liquids (ILs) properties can generate soft materials with potential interest in different areas. It is important to emphasise that, while ILs and LCs are well-researched, ILCs accounted for only 2% of the publications on these three topics in 2000, increasing to just 4% after 22 years, which suggests that few researchers are working at the intersection of liquid crystals and ionic liquids fields. However, this does not mean that the two areas do not merge, and it certainly deserves further investigation.

Regarding the phase structure, in general, ionic liquid crystals containing various organic cations, such as imidazolium, pyridinium, ammonium and phosphonium, combined with halide anions tend to show smectic phases. This contrasts with conventional liquid crystals, which exhibit other liquid crystalline phases. Therefore, designing different cation–anion combinations to explore other types of ILCs, including nematic, cubic and columnar phases, seems interesting. It is also observed that replacing halides with organic anions contributes to the preparation of ionic liquids without mesophases.

Given the number of applications involving liquid crystals and the unique properties of ionic liquids, new uses for ILCs as advanced materials are anticipated. In this context, stimuli-responsive ionic liquid crystals, namely the photo-, electro- and thermochromic ones, are promising for both academic and industrial purposes. Furthermore, considering the already proven relevance of confining liquid crystals, the confinement of ILCs is another topic that deserves attention, as it enables the rational design of electro-optical devices based on ionic liquid crystals. Other research areas, such as catalysis, energy and pharmaceuticals, should be investigated for future applications of these organic salts. 

## Data Availability

No new data were created or analysed in this study. Data sharing is not applicable to this article.
